# Review: Dietary fiber utilization and its effects on physiological functions and gut health of swine

**DOI:** 10.1017/S1751731115000919

**Published:** 2015-05-22

**Authors:** R. Jha, J. D. Berrocoso

**Affiliations:** Department of Human Nutrition, Food and Animal Sciences, College of Tropical Agriculture and Human Resources, University of Hawaii at Manoa, Honolulu, HI 96822, USA

**Keywords:** dietary fiber, digestion, fermentation, gut health, pig

## Abstract

Although dietary fiber (DF) negatively affects energy and nutrient digestibility, there is growing interest for the inclusion of its fermentable fraction in pig diets due to their functional properties and potential health benefits beyond supplying energy to the animals. This paper reviews some of the relevant information available on the role of different types of DF on digestion of nutrients in different sections of the gut, the fermentation process and its influence on gut environment, especially production and utilization of metabolites, microbial community and gut health of swine. Focus has been given on DF from feed ingredients (grains and coproducts) commonly used in pig diets. Some information on the role DF in purified form in comparison with DF in whole matrix of feed ingredients is also presented. First, composition and fractions of DF in different feed ingredients are briefly reviewed. Then, roles of different fractions of DF on digestion characteristics and physiological functions in the gastrointestinal tract (GIT) are presented. Specific roles of different fractions of DF on fermentation characteristics and their effects on production and utilization of metabolites in the GIT have been discussed. In addition, roles of DF fermentation on metabolic activity and microbial community in the intestine and their effects on intestinal health are reviewed and discussed. Evidence presented in this review indicates that there is wide variation in the composition and content of DF among feed ingredients, thereby their physico-chemical properties in the GIT of swine. These variations, in turn, affect the digestion and fermentation characteristics in the GIT of swine. Digestibility of DF from different feed ingredients is more variable and lower than that of other nutrients like starch, sugars, fat and CP. Soluble fractions of DF are fermented faster, produce higher amounts of volatile fatty acid than insoluble fractions, and favors growth of beneficial microbiota. Thus, selective inclusion of DF in diets can be used as a nutritional strategy to optimize the intestinal health of pigs, despite its lower digestibility and consequential negative effect on digestibility of other nutrients.

## Implications

This review provides relevant information on the utilization of dietary fiber (DF) by pigs and its effects on gut physiological functions, microbiota and health. This review delivers in-depth insight on both negative and positive effects of different fibers inclusion in swine diets. The DF lowers nutrient digestibility in swine. But, the fermentation of DF in the gut affects positively by modulating gut environment and potentially favoring ‘beneficial bacteria’, thereby improving gut health of pigs. These insights will help swine nutritionists and researchers in nutrition programing for better gut health of pigs by utilizing dietary fiber from different sources.

## Introduction

During the last half century, there has been tremendous development in the field of pork production, resulting to more than 109 million tons of pork produced per year (FAO, 2013). This progress has been achieved by an intensification of the swine production systems, coupled with selective breeding programs and a better knowledge of pig nutrition. Feeding strategies based on antibiotics used as growth promoters aimed at improving the pig growth rate by improving its gut health status. However, there is a growing concern about the resistance of numerous bacteria to antibiotics used in human medicine, and is claimed to be due to the consumption of meat from animals grown with in-feed antibiotics, as meat from antibiotic-fed animals were found to have antibiotic-resistant bacteria (Gebreyes *et al.*, 2006). Dietary fiber (DF) has been found to be an effective alternative to growth promoters (Verstegen and Williams, 2002), to improve gut health (Williams *et al.*, 2001) by modulating gut microbiota, improve growth performance and reduce post-weaning diarrhea of the pigs (Mateos *et al.*, 2006).

In commercial pig production, plant carbohydrates (CHO) represent the main fraction of a pig diet, accounting for more than 2/3^rd^ of the dry matter (DM; Bach Knudsen, 1997) and the single most abundant feed energy in diets for piglets, growing pigs and sows comprising 60.0% to 70.0% of total energy intake (Bach Knudsen *et al.*, 2012). However, part of the CHO is not digested by the digestive enzymes of the small intestine and becomes available as a substrate for bacterial fermentation, mainly in the large intestine. This fraction, that is, DF, reduces nutrient and energy digestibility (Bach Knudsen, 2001; Noblet, 2007; Jha *et al.*, 2010). Its physico-chemical properties (like solubility, viscosity and water-holding capacity (WHC)) also have a marked effect on nutrient digestibility along the gastro-intestinal tract (Bach Knudsen and Hansen, 1991; Chabeauti *et al.*, 1991; Molist *et al.*, 2014).

Despite its negative impact on nutrient and energy digestibility (Bach Knudsen, 2001; Noblet, 2007; Jha *et al.*, 2010), there is growing interest to include DF in pig diets, due to its possible effects on gut health, welfare and environment. Moreover, during the last decade, there has been dramatic shift in the landscape of the feed industry, in terms of price and availability of feed ingredients for animal feeds. There is increased availability of different alternative feed ingredients and coproducts from distillers and milling industries, which are rich sources of DF as well. However, it is important to know the implication of the use of these relatively new and potential DF sources in pig nutrition. Therefore, nutritionists are attempting to gain a more thorough understanding of inclusion of DF in pig diets.

To address the concerns and have a better understanding of DF and its role, this review has analyzed different aspects of DF in swine nutrition. More specifically, composition of DF in different feed ingredients (grains and coproducts) is first briefly reviewed. The digestibility and fermentability of the DF components in the gastrointestinal tract (GIT) of pigs and their effect on gut physiology, microbial environment and health are highlighted.

## DF

### DF and its components

The term ‘dietary fiber’ was first used by Hipsley in 1953 (De Vries *et al*., 1999) for ‘the non-digestible constituents that make up the plant cell wall’. However, different definitions have been proposed and used over time. It is now accepted that an accurate definition of DF must include the physiological effects of fiber. Therefore, an important aspect of the definition is that DF consists of CHO that are indigestible by endogenous animal enzymes (AACC, 2001).

Broadly, DF includes cell wall compounds like cellulose, hemicelluloses, mixed linked *β*-glucan (*β*G), pectins, gums and mucilages (Davidson and McDonald, 1998). Lignin, a complex phenolic compound, is also included in DF because it is a constituent of the plant cell walls that can greatly affect the digestibility of plant-derived foods (Theander *et al.*, 1989). From a physiological point of view, non-starch polysaccharides (NSP), non-digestible oligosaccharides and resistant starch (RS) have to be included in the soluble DF fraction because they are not hydrolyzed by endogenous enzymes, and consequently, become available as substrates for microbial fermentation in the intestine (Cummings and Stephen, 2007).

### Sources of DF and interest for their use in swine diet

The origin and composition of DF could be responsible for large variations in their utilization (Chabeauti *et al.*, 1991). The physico-chemical properties of the DF sources may lead to changes in the gut environment, altering the growth of the gut microflora. The acceptability of the alternative feed ingredients in pig diets depends on several factors, like the DF content, the degree of microbial fermentation in the large intestine and the extent of absorption and utilization of the volatile fatty acid (VFA) produced (Molist *et al.*, 2014). The fiber sources are fermented in the GIT producing VFA, which in turn positively affects gut health (Lindberg, 2014). Wellock *et al.* (2007) noted that gut health might benefit most from diets containing appropriate sources of predominantly soluble NSP rather than insoluble NSP. Soluble DF includes pectins, *β*G, gum and hemicelluloses, while cellulose and lignin compromise the insoluble fraction (Davidson and McDonald, 1998). Thus, it is imperative to know the source and type of fiber being supplied in pig diets.

There is also increasing interest and incentive for the identification and characterization of alternative feed ingredients. These alternative feed ingredients include cereals and legume grains, distillery coproducts, coproducts from oil industry (like canola meal) and wheat flour milling (millrun and bran) and other fibrous feeds.

Cereal grains and their coproducts account for the major part of pig rations as main sources of energy. The DF of cereal grains are mainly composed of NSP (arabinoxylans, **AX**, *β*G and cellulose) and noncarbohydrate component lignin (Bach Knudsen, 2014). In addition, small amounts of pectin substances found in the stems and leaves of cereals (Choct, 1997). Several workers mentioned that the level of DF in the commonly available feed ingredients vary in relation to type and quality. As a reference to show the variation found in fiber components, the composition of some of the most common cereals and coproducts is presented in Table 1. Rye, wheat, corn and sorghum are all rich in AX, whereas barley and oats contain a high level of *β*G. The AX from rye and wheat and *β*G from barley and oats are to a large extent soluble, whereas the solubility of AX found in corn and sorghum is lower than the other cereals (Bach Knudsen *et al.*, 2014).

**Table 1:** Types and levels of fiber components in some common cereal grains and coproducts (g/kg DM)

		Barley^2^ ^,^ ^3^ ^,^ ^4^		Oats^2^ ^,^ ^4^								
	Wheat^1^	Hulled	Hulless	Hulled	Hulless	Corn^1^	Sorghum^1^	Rye^2^	Wheat bran^2^	Wheat middlings^1^	DDGS^1^	Sugar beet pulp^5^
Starch	618	587	645	468	557	620	690	613	222	168	86	5
Cellulose	13	39	10	82	14	17	15	15	72	67	58	203
NCP												
Soluble	19	56	50	40	54	25	4	42	29	12	34	290
Insoluble	62	88	64	110	49	38	47	94	273	227	158	207
NSP												
Arabinoxylans	81	12	48	98	36	17	17	89	238	52	61	165
*β*-glucan	8	43	42	28	41	6	1	20	24	21	63	8
Mannose	2	2	4	3	3	2	1	3	5	19	9	8
Galactose	3	2	3	7	4	8	3	3	9	13	14	38
Uronic acids	4	2	2	10	5	8	4	2	15	16	16	199
Total NSP	95	167	124	232	116	81	66	132	374	250	192	700
Lignin	18	35	9	66	32	8	16	21	75	39	32	37
Dietary fiber	112	202	133	298	148	89	83	153	449	289	322	737

DM=dry matter; NCP=non-cellulosic polysaccharides; NSP=non-starch polysaccharides; DDGS=distillers dried grains with solubles.

1 According to Jaworski *et al.* (2015).

2 According to Bach Knudsen (1997).

3 According to Holtekjolen *et al.* (2006).

4 According to Jha *et al.* (2011a).

5 According to Serena and Knudsen (2007).

Corn coproducts are typically rich in DF but variable in starch, amino acid and fat. The concentration and composition of DF of feed ingredients is important, because it may reduce amino acid and energy digestibility (Noblet and Perez, 1993). The insoluble fraction of DF in corn and its coproducts is resistant to fermentation (∼40% is fermented in the entire GIT of the pigs), consisting of insoluble NSP such as cellulose, arabinoxylans and lignin (Bach Knudsen, 1997). Increasing the efficiency of starch and oil extraction from the corn grain, resulting in changes in chemical composition of corn coproducts, present a challenge to estimate their nutritional value (Gutierrez *et al.*, 2014). In fact, Fairbairn *et al.* (1999) reported that NDF or ADF alone accounted for 68.0% and 85.0% of the total variation in digestible energy content of barley, respectively.

Wheat (*Triticum aestivum*) is primarily used in pig diets as source of energy. However, it contains significant amount of DF, including AX, *β*G and cellulose. There are several wheat varieties, which vary widely in the amount and type of DF content. For example, in 12 wheat cultivars studied, ADF and NDF content ranged from 1.8% to 3.2% and 7.2% to 9.1%, respectively (Jha *et al.*, 2011c). Similarly, Kim *et al.* (2005) reported wide variation in the NSP and lignin contents of wheat while reviewing 426 samples dataset. The NSP content varied from 3.5% to 10.6% AX, 0.3% to 1.2% *β*G and 7.5% to 16.6% total NSP and the lignin content ranged from 0.9% to 1.1%. This variation in amount and type of DF content of wheat can be attributed to different varieties (genetic makeup), growing locations and climate.

Barley (*Hordeum vulgare* L.) grains are relatively rich in DF such as *β*G, cellulose and AX. In particular, hulless barleys contain high levels of soluble *β*G (Izydorczyk *et al.*, 2000), which are associated with health benefits for the gut (Brennan and Cleary, 2005). Even within hulless barleys, the variability in total DF, soluble and insoluble NSP is very wide (Holtekjolen *et al.*, 2006; Jha *et al.*, 2011b). There is also wide variation in *β*G content within and between cereal types, ranging from ∼1.9% to 11.0% in barley, 1.7% to 7.0% in oats and 0.2% to 0.7% in wheat (Lee *et al.*, 1997; Brennan and Cleary, 2005; Jha *et al.*, 2011b). The rate of solubility of *β*G also varies, for example, Lambo *et al.* (2005) found that most of the *β*G in barley fiber were insoluble (about 85.0%) whereas in oat, the opposite is observed, where about 68.0% of *β*G were found in soluble fiber fractions.

Oat (*Avena sativa* L.) contains both soluble and insoluble DF, with high levels of *β*G, AX and cellulose (Johansen *et al.*, 1997; Jha *et al.*, 2011b). Among the DF components of oat, *β*G play an important role because of their functional properties in the GIT (Pieper *et al.*, 2008; Jha *et al.*, 2010).

Grain legumes are used in pig diets primarily as sources of protein and contain significant amounts of NSP as well. Cellulose and xylans are found in the hulls, whereas pectic polysaccharides are found in the cotyledons (Choct, 1997; Jha and Leterme, 2012).

The wheat flour milling industry generates a number of coproducts (wheat bran (WB), millrun, middlings, shorts, etc.), which are classified based on their DF content (Jha *et al.*, 2012). These coproducts contain much higher DF than wheat as are enriched during processing of wheat to produce flour. In the six types of wheat flour milling coproducts studied, ADF and NDF content varied from 8.0% to 15.5% and 22.9% to 49.2%, respectively (Jha *et al.*, 2012).

Distillers dried grains with solubles (DDGS) is a coproduct from dry mill ethanol plants resulting from the fermentation of starch of cereal grains (corn, wheat, etc.) to produce fuel ethanol and carbon dioxide. Cereal grains are good source of starch, and as most of the starch is converted to ethanol during fermentation, which results in increased concentrations of the protein, oil and fiber that are two to three times higher than in the parent grain. Because of the depletion of most of the starch, the concentrations of CHO is lower than in the parent grain (Widmer *et al.*, 2007) with most of the CHO present as fiber. The concentration of the different fiber fractions (NDF and ADF) and total DF is approximately three times greater in DDGS than in parent grains. However, the nutrient composition of DDGS varies depending on the ethanol plant where they are processed and the DDGS originating from different parent grains (Pedersen *et al.*, 2014; Jha *et al.*, 2015). The digestibility of fiber in DDGS is <20.0% in the small intestine and <50.0% over the entire GIT, and the DF, therefore, contributes relatively little to the energy value of these products. Moreover, low digestibility of DF in distillers coproducts results in increased quantities of manure being excreted from pigs fed these products, and the overall DM digestibility of diets containing distillers coproducts is lower than in corn-based diets (Pedersen *et al.*, 2007). This lower digestibility of DDGS can be attributed to the complex fiber–starch–protein matrix (Jha *et al.*, 2015), which limits the accessibility and action of endogenous enzymes for degradation. However, supplementation of exogenous multi-carbohydrase and protease enzymes may enhance the degradation of DDGS, thereby improving the nutrient release (Jha *et al.*, 2015; Pedersen *et al.*, 2015).

Canola meal is a coproduct derived after oil extraction processing, and can be used as ingredients in animal diets. CHO in canola seed may be categorized into soluble sugars and oligosaccharides, insoluble CHO and fiber. The concentration of soluble CHO in mature seeds is ∼10.0% of the oil-free weight, with sucrose ranging from 3.9% to 9.8%, raffinose from 0.3% to 2.6%, stachyose from 0.8% to 1.6%, fructose from 0.1% to 0.5% and glucose from 0.1% to 0.4% (Barthet and Daun, 2011). The concentration of hemicellulose is ∼3.0%, cellulose ranges from 4.0% to 5.0%, and starch is ∼1.0% (Salunkhe *et al.*, 1992). The concentration of crude fiber, NDF and ADF in canola meal ranges from 10.0% to 12.0%, 22.0% to 30.0% and 15.0% to 20.0%, respectively. Canola meal has relatively high concentration of DF because hulls in canola seeds stay with the meal (Barthet and Daun, 2011). However, canola breeding programs have developed canola varieties with greater oil and protein content than traditional varieties. The new high-protein varieties of canola also contain less DF, and the resulting canola meal, therefore, has a reduced DF concentration compared with conventional canola.

## DF and digestion

### Effect of DF on digestion and physiological functions in gut

#### Effect of DF on digestibility

Digestibility of DF is more variable (40.0% to 60.0%) and lower than that of other nutrients like starch, sugars, fat and CP (above 80.0% in general). It is negatively affected by the amount and source of DF content in the diet (Noblet, 2007; Jha *et al.*, 2010). Consequently, the digestible energy content of diets is negatively and linearly affected by DF (Noblet, 2007). DF is better digested in adult sows than in growing pigs. The difference reaches 0.6 MJ/kg DM, on average (Noblet, 2007). This is ascribed to differences in the physiological stage of pigs as there is a higher rate of degradation of DF in the hindgut of sows, compared with growing pigs, due to longer retention time consecutive to their higher GIT volume, combined with a lower feed intake per live weight (Le Goff *et al.*, 2002). However, it is not known if the DF in all cereal grains has similar effects on the digestibility of energy and nutrients in the diet. Just *et al.* (1983) found that every 1.0% of additional crude fiber in the diet decreases the gross energy digestibility by 1.3% and metabolizable energy by 0.9%. The NSP, both in purified form and embedded within the matrix, also reduce CP digestion in pigs (Bedford *et al.*, 1992; Jha *et al.*, 2010). There is a linear decrease in apparent ileal digestibility of DM and CP with increased levels of purified NDF in the diet (Schulze *et al.*, 1994) and lower organic matter, CP and starch digestibility in diets containing hulled barley, as compared with hulless barley-based diets in pigs. Also, Jha *et al.* (2010) reported that the lower organic matter and starch digestibility of the hulled barleys and oats was likely due to greater insoluble DF content, which negatively affects accessibility and the action of endogenous enzymes required for insoluble DF digestion in the upper gut and microbial fermentation in the lower gut.

On the other hand, the inclusion of the rice in the diet improves the digestibility of nutrients as compared with other cereal grains (Mateos *et al.*, 2006; Cervantes-Pahn *et al.*, 2014). These improvements in nutrient digestibility, especially gross energy, organic matter, starch and total CHO in rice diet, indicate that in young pigs, energy and nutrients from rice are better digested and absorbed than energy and nutrients from corn (Solá-Oriol *et al.*, 2010).

The digestibility of nutrients in sorghum and wheat is relatively similar to that of corn, but in terms of grain structure and nutrient composition, sorghum is more similar to corn than to wheat (Taylor and Emmambux, 2010). But, CP digestibility of sorghum is less than corn. The reduced digestibility of CP in sorghum could be attributed to the binding of tannins to the protein in sorghum, which makes the protein resistant to proteolysis (Duodu *et al.*, 2003). However, tannins content of the sorghum varies depending on the varieties and parts of the sorghum grain. Moreover, Wilfart *et al.* (2007b) reported decreasing apparent total tract digestibility of DM, organic matter, CP and gross energy when added 0%, 20% and 40% WB (16.5%, 20.9% and 27.0% total DF, respectively) to a wheat–barley–soybean meal diet. These authors suggested that digestion of the WB in the hindgut is affected by the time that the digesta is exposed to fermentation, and a rapid passage of digesta may reduce the efficacy of the digestion process. In fact, WB is one of the most effective fiber sources for increasing the rate of passage in the digestive tract.

#### Effect of DF on physiological functions

The presence of DF in the diet does not only affect digestibility but also other physiological functions in the gut. The latter are affected by the level and type of fiber (Schulze *et al.*, 1995) and their physico-chemical properties, like WHC, solubility and viscosity (Leterme *et al.*, 1996; Molist *et al.*, 2014).

The presence of soluble DF in the diet increases digesta viscosity (Gallaher *et al.*, 1999) and increased viscosity in the digesta can limit the interaction between nutrients and enzymes facilitating the formation of an unstirred water layer in the intestinal surface, thereby creating a physical barrier and consequently, reducing nutrient digestion and absorption. Moreover, insoluble DF sources such as WB are relatively resistant to microbial degradation (Jorgensen *et al.*, 1996) and its inclusion in the diet produces an increase in fecal DM and bulkiness (Wilfart *et al.*, 2007b). On the other hand, DF inclusion in the diet increases the endogenous nitrogen losses depending on DF level, type (Schulze *et al.*, 1995) and physico-chemical properties such as WHC (Leterme *et al.*, 1996). For example, soluble DF increases digesta viscosity and endogenous nitrogen losses (Mariscal-Landin *et al.*, 1995). The high viscosity of the gut chyme stimulates the epithelial cell proliferation and may contribute to some loss of epithelial cells (Gee *et al.*, 1996). In weanling pigs, Schiavon *et al.* (2004) reported that an increase in intestinal viscosity increased cell exfoliation in the apical part of the intestinal villus, causing atrophy and deeper crypt depth. Consequently, an increase in intestinal viscosity might reduce the digestion and absorption of nutrients in the diet (Molist *et al.*, 2014). In this respect, Lizardo *et al.* (1997) reported decreasing apparent ileal digestibility in 25-day-old piglets fed a control diet or a same diet supplemented with 12.0% sugar beet pulp (SBP). In presence of DF, there is also an increase in the pancreatic secretions and the number of goblet cells (Schneeman *et al.*, 1982). Moreover, there is increase in mucus secretion in the small intestine (Mariscal-Landin *et al.*, 1995), which might be due to the mechanical effect of DF on the gut wall that affects the integrity of the mucus layer, resulting in superficial cell lesions (Schmidt-Willig *et al.*, 1996). However, Leterme *et al.* (1998) did not observe any influence of insoluble NSP with high WHC on protein digestion and absorption, as opposed to soluble NSP with high WHC. In the study, the contrasted result of the effect of NSP on endogenous nitrogen losses was due to different sources of fibers used, which supports the findings that the source of DF with different physico-chemical properties behave differently and have different effects on nutrient digestibility and physiological properties in the gut.

It can be concluded that DF content negatively affects nutrient and energy digestibility, which varies according to the amount and source of DF and their physico-chemical properties. Moreover, different types of DF also exert their effect on different physiological functions in the gut.

## Degradability of DF in the upper and lower gut

### Degradability of DF in the upper gut

DF escapes enzymatic digestion in the small intestine and becomes available for fermentation by bacteria in the colon. However, substantial degradation of DF may also occur in the small intestine (Jensen and Jorgensen, 1994; Jha *et al.*, 2010; Jha and Leterme, 2012). Fiber-degrading bacteria are present in the stomach and in the proximal small intestine. They can partially disrupt the cell wall components of fiber, which leads to partial digestion (Varel and Yen, 1997). Bach Knudsen *et al.* (2008) summarized the ileal digestibility of NSP and their components from different studies. The results clearly indicate a wide variation in the digestion of NSP components, within and between different cereal sources. The wide variation in the fiber degradability can be ascribed to the physico-chemical properties of DF, the complexity of digestion/fermentation process, differences in experimental design, sample collection and analytical techniques. Gdala *et al.* (1997) reported lower digestibility of xylose, arabinose and uronic acids in the small intestine of piglets compared with glucose when fed diets based on cereals and soybean meal. This might be due to the high digestibility of mixed linked *β*G, which is highly degradable in the upper gut, due to its soluble nature (Bach Knudsen and Hansen, 1991; Jha *et al.*, 2010, 2011b). Also, Jha and Leterme (2012) suggested that the higher insoluble DF content of the different fiber sources such as WB or DDGS affects negatively to the accessibility and action of the endogenous enzymes in the upper gut and microbial fermentation in the lower gut, resulting in lower degradability of DF.

### Degradability of DF in the lower gut

There is wide variation in the degradation of fiber in the large intestine, ranging from 48% to 95.0% (Bach Knudsen *et al.*, 1993a; Jha *et al.*, 2010; Jha and Leterme, 2012). Similarly, the total tract apparent digestibility of cellulose varies widely (2.0% to 84.0%). Soluble pectin and hemicelluloses are digested to a greater extent than cellulose, while soluble *β*G from barley are almost completely digested by the end of the gut; the prececal digestibility has been found to range as high as 70.0% to 97.0% (Bach Knudsen *et al.*, 1993a; Jha *et al.*, 2010). On the other hand, insoluble branched-chain AX from wheat, rye and oat are less digestible in the pig gut (Bach Knudsen and Hansen, 1991; Glitsø *et al.*, 1998). There are also noted effects of the DF source on variation in NSP digestibility. Chabeauti *et al.* (1991) found that the NSP digestibility in growing pigs varies from 16.3% for wheat straw, 43.5% for WB, and 69.5% for SBP to 79.1% for soybean hulls. The poor digestibility of WB is ascribed to their high lignin content that makes the NSP less fermentable compared with highly digestible pectin substances of SBP and soybean hulls (Karr-Lilienthal *et al.*, 2005).

Gdala *et al.* (1997) analyzed the digestibility of different NSP residues in piglets and found that the rate and overall degradation of the polymers in the large intestine was largely influenced by the chemical nature of the DF, especially its solubility and degree of lignification. Similar results were obtained by Johansen *et al.* (1997) while studying the degradation of *β*G and AX from oat bran in the pig gut. However, the total loss of NSP from the anterior to terminal ileum was lower than reported by other workers in old pigs (Bach Knudsen and Hansen, 1991; Bach Knudsen *et al.*, 1993a), possibly due to lower microbial activity in young piglets. Among the NSP components, soluble *β*G, AX and pectins are rapidly degraded in the cecum and proximal colon while insoluble components of NSP like cellulose and insoluble AX are degraded slowly and at the distal part of the colon (Bach Knudsen *et al.*, 1993a; Glitsø *et al.*, 1998; Canibe and Bach Knudsen, 2002). In this respect, Gidenne (2015) mentioned that the amounts of DF entering the cecum of rabbit is not a limiting factor for the fermentation processes, because the digesta retention time in the cecum of rabbit is very short as compared with pigs, and consequently, soluble fiber fractions such as pectins are degraded easily. Moreover, soluble and non-cellulosic mannose and galactose are highly digestible and fermentable compared with the insoluble cellulosic components of NSP (Serena and Knudsen, 2007).

## Fiber fermentation in the GIT

### Fiber fermentation

The susceptibility of DF to microbial fermentation varies depending on the accessibility of DF to the microbial population in the hindgut (Oakenfull, 2001). In monogastric animals, the large intestine is the most important site of fermentation (Williams *et al.*, 2001). Fermentation of soluble DF is mainly at the proximal colon, whereas fermentation of insoluble DF is sustained until the distal colon (Choct, 1997). However, substantial fermentation of soluble DF has been observed in the pig’s small intestine (Jensen and Jorgensen, 1994; Jha *et al.*, 2010; Jha and Leterme, 2012). Fiber fermentation is an extremely complex process, affected by many factors in the GIT, including the host, its microflora and their interaction, which takes place between them (Williams *et al.*, 2005).

#### Fiber fermentation and production of metabolites

Fermentation of DF is more variable than digestion of the macronutrients starch, fat and CP (generally above 80.0%; Bach Knudsen *et al.*, 2008). The variation in fermentability is mainly due to changes in physico-chemical properties of DF such as bulk, viscosity, solubility, WHC and fermentability. DF fermentation results in the production of VFA like acetate, propionate and butyrate, along with some gases like hydrogen, carbon dioxide and methane (Macfarlane and Macfarlane, 1993; Williams *et al.*, 2001). Acetate is the most abundant VFA, comprising about 60.0% of the total VFA produced in the hindgut, whereas propionate and butyrate are produced in smaller quantities (Lunn and Buttriss, 2007). However, the extent of fermentation and the profile in VFA depend on the substrate (Salvador *et al.*, 1993; Jha *et al.*, 2012) while the rate of fermentation of DF in the pig’s intestines depends on its composition and physico-chemical properties, degree of lignification and particle size (Le Goff *et al.*, 2003) and transit time in the digestive tract (Wilfart *et al.*, 2007a). Soluble DF has, in general, a higher WHC than insoluble DF that give raise to a larger surface area and thereby large areas for bacterial enzyme attack. Thus, these characteristics are directly dependent on the botanical origin and (or) processing of the DF source (Johansen *et al.*, 1997).

In addition to VFA, other metabolites such as lactate, ethanol and succinate are also produced from bacterial fermentation of DF (Drochner *et al.*, 2004). The majority of these metabolites (possibly except ethanol) are further converted into VFA by cross-feeding mechanisms (Macfarlane and Gibson, 1995).

#### Fate of fermentation metabolites in the pig large intestine

The main site of VFA absorption in pigs is the large intestine (Imoto and Namioka, 1978) where the majority (about 90.0%) of the VFA are absorbed and metabolized (Jorgensen *et al.*, 1997).

The VFA are absorbed in the gut through passive diffusion. The exact mechanism for absorption is still unclear. However, several mechanisms have been proposed, stating their dependence on luminal pH, CO_2_, as well as the fluxes of water, protons and inorganic ions (Bugaut, 1987). The fermentation metabolites are taken up by the cells in the intestines and used for bacterial growth (Bach Knudsen, 2001). Although VFA are primarily taken up and metabolized by colonocytes, these are also used as a source of energy by other tissues (Wong *et al.*, 2006). The absorbed VFA are basically metabolized in three major sites: in the ceco-colonic epithelial cells that use butyrate for their energy production pathway; liver cells that metabolize residual butyrate and propionate for gluconeogenesis, as well as 50.0% to 70.0% of acetate; and muscle cells, mainly from skeletal and cardiac muscles that oxidize the residual acetate (Roberfroid, 2007). The energy produced from VFA may contribute up to 15.0% of the maintenance energy requirements of growing pigs (Dierick *et al.*, 1989) and even up to 30.0% in gestating sows (Varel and Yen, 1997).

Absorption of VFA also facilitates absorption of other nutrients from the diet. Water and sodium are absorbed along with VFA (Yen, 2001). Plant lignans, diphenolic compounds similar to endogenous steroid hormones, are also co-transported by VFA (Bach Knudsen *et al.*, 2006).

### Effect of source of fiber on metabolite production

Among the non-digestible oligosaccharides, fructo-oligosaccharides (FOS) are the most extensively studied. The FOS contains 2–70 fructose residues and Bifidobacteria can digest them as they produce the enzyme fructofuranosidase (Gibson *et al.*, 2004). FOS are fermented by bacteria, yielding energy for bacterial growth. Houdijk *et al.* (2002) evaluated the effect of FOS and transgalacto-oligosaccharides (TOS) in comparison with non-fiber control diets in weaned pigs and found that both FOS and TOS increased VFA production in the gut. However, there were differences in the concentration of VFA in different sections of the gut between FOS and TOS fed pigs, which supports the view that FOS and TOS have different fermentation characteristics in the GIT of the pigs.

Among the DF fractions, *β*G is gaining more attention as it is a source of easily fermentable energy for intestinal microbiota. It yields higher levels of VFA (Brennan and Cleary, 2005) due to a relatively high concentration, soluble state and high molecular weight and results in several beneficial physiologic effects to the host (Dongowski *et al.*, 2002). Oat bran, a rich source of soluble DF in the form of *β*G, produces almost twice as much VFA per gram DF as WB in the pig intestines (Bach Knudsen and Hansen, 1991; Bach Knudsen *et al.*, 1993a). However, AX and not *β*G in the cell walls of oat bran are responsible for the enhanced butyric acid production of oat bran (Bach Knudsen *et al.*, 1993b). Bach Knudsen and Canibe (2000) found higher concentrations and flows of lactic acid in the ileum of cannulated pigs after feeding a diet supplemented with soluble DF from oat bran, which supports the view that *β*G stimulates the production of lactic acid in the small intestine, which is found to promote the development of Lactobacilli, a family of health-promoting bacteria.

There are noted effects of DF on VFA production and profile, depending on type and source of DF. For example, Freire *et al.* (2000) compared the effects of inclusion in the diet of 20.0% of WB, SBP, soybean hulls or alfalfa meal on concentration of the total VFA in the cecum and reported that the inclusion of soybean hulls in the weaning diet increased the concentration of total VFA by 11.2%, 30.5% and 27.2% as compared with WB, SBP and alfalfa diets, respectively. The values suggests that soybean hulls is highly degraded in the cecum, and is in agreement with the high digestibility values of the NDF and ADF fractions reported in this research. Also, the lower values of VFA measured in the SBP diet might also be associated with a higher absorption rate of its metabolites in the cecum. Carneiro *et al.* (2008) compared the effect of two fiber sources, WB and maize cobs in weaned pigs and found no difference in the amounts of VFA in the small intestine. However, there was higher acetic acid and lower butyric acid production in the cecum when WB was replaced with maize cobs. Findings of these studies clearly indicate that not only the amount and type of substrate, but also the source of fiber fraction is important to determine the amount and type of VFA production.

### Effect of fiber fermentation on gut microbiota

The influence of diet on microbial communities in the pig intestines has been of interest for long time. However, the interaction of diet and microbiota in the intestines of the pig are still not well understood.

The GIT microbiota of pig is composed primarily of bacteria. The microbial population increases from 10^3^ to 10^5^/g of digesta in the stomach to 10^9^ to 10^10^ in the distal small intestine, and further to 10^10^ to 10^11^ in the large intestine of pigs, belonging to more than 50 genera and over 500 species of bacteria (Jensen and Jorgensen, 1994; Gaskins, 2001). The majority (about 90.0%) of the cultivable bacteria are Gram-positive, strict anaerobes belonging to the *Streptococcus*, *Lactobacillus*, *Eubacterium*, *Clostridium* and *Peptostreptococcus* genus while the remaining 10.0% of total flora belongs to Gram-negative of *Bacteroides* and *Prevotella* groups (Gaskins, 2001). Each bacterial species occupies a particular niche with numerous interrelationships between them (Flint *et al.*, 2008).

#### Effect on microbial composition

The population and activity of bacteria in the gut is influenced by several factors, the main one being diet (Bach Knudsen *et al.*, 2012). More specifically, the structure and composition (Konstantinov *et al.*, 2004; Bindelle *et al.*, 2010), solubility (Hogberg and Lindberg, 2004) and amount and type of substrate available (Macfarlane and Macfarlane, 1993) affects the gut microbial ecology. Among the different constituents of diets, DF is found to affect the gut environment (Awati *et al.*, 2005; Jha *et al.*, 2010; Jha and Leterme, 2012). The source of DF affects the digestion site and gut environment, thereby affecting the conditions for the proliferation of microbiota in the gut (Hogberg and Lindberg, 2004). Moreover, DF serves as an energy source for microbes and supports their proliferation. Gidenne (2015) reported that the energy provided by the cecal VFA could reach up to 50% of the maintenance energy in growing rabbit. In pigs, Bach Knudsen *et al.* (1991) reported 5.5 times (as measured by ATP concentration) increased microbial activity in the GIT of pigs when fed with a high-fiber diet. In addition, there was increased (five to nine times) carbon dioxide and methane production, suggesting increased microbial fermentation that takes place in the GIT of pigs fed a high-fiber diet. Similar increased microbial activity was observed in the intestines of pigs fed pea fiber and pectin, as indicated by higher bacterial counts, ATP concentration, adenylate energy charge and low pH (Jensen and Jorgensen, 1994). However, Varel *et al.* (1982) noted that there was initially a decrease in the bacterial population of the pig intestines when the animals were fed with high-fiber diet (50.0% alfalfa meal) in lean genotype pigs. The microbial population, however, increased after continuous fiber-feeding for 17 weeks. It suggests that there is some kind of adaptation of the microbiota in the pig intestines when fed with high DF diets.

DF affects fermentation in the GIT by stimulating the growth or metabolism of special bacterial species (Williams *et al.*, 2001). These increased numbers of cellulolytic bacteria enhance the hindgut fermentation and production of VFA, which decreases the pH of the gut content. A decrease in pH promotes growth of beneficial bacteria (e.g. *Bifidobacteria* spp., *Lactobacilli* spp.), at the expense of pathogenic ones like *Clostridium* or *Salmonella*, which contribute to enhance the health of host species (Bouhnik *et al.*, 2004). This phenomenon is termed as ‘prebiotic effect’ (Gibson and Roberfroid, 1995).

The potential ‘prebiotic effect’ of DF has been studied in several monogastric species, including swine. The results are quite variable from one study to another in terms of their effect on microbial population, diversity and gut health, which can be ascribed to the type of substrate available for fermentation and the gut environment of the host. At the increased level of DF in the large intestine, there is an increase in activity of the entire microbial community. However, some types of DF may have selective effects and stimulate particular niches of microorganisms (Louis *et al.*, 2007).

Estrada *et al.* (2001) found increased numbers of *Bifidobacteria* spp. and decreased numbers of total anaerobes and *Clostridia* in feces of pigs fed with diets containing 0.5% FOS in conjunction with *Bifidobacterium longum*. Drew *et al.* (2002) compared the effect of CHO sources (corn, wheat and barley) in weaned pigs and found that the bacterial population was significantly related with ADF and NDF contents of the diets. There were increased *Lactobacilli* spp. and decreased *Enterobacteria* spp. populations in barley-fed pigs, as compared with corn-fed pigs. Moreover, barley-based diets increased *Lactobacilli* spp. and *Bifidobacterium* spp. in the cecum, compared with corn-based diets. One possible explanation might be the higher amount of *β*G in barley-based diets, compared with corn-based diets, which is found to enhance the growth of beneficial bacteria at the cost of harmful bacteria. In fact, Pieper *et al.* (2008) reported that dietary *β*G in barley-based diets increases colonic *lactobacilli* spp. and *bifidobacteria* spp. promoting butyrate-producing bacteria. Interestingly, wheat-based diets had higher numbers of *Bifidobacterium* spp. and lower numbers of total aerobes and *Clostridium* spp., compared with barley-based diets. Similarly, Nielson *et al*. (2014) reported that pigs fed with diet rich in AX resulted in higher levels of *Bifidobacterium* spp. and *Lactobacillus* spp. in the feces. These can be ascribed to the complexity of the various bacterial species and fermentable substrates present, especially the monomers of the fiber, which are selective for certain microbes. Konstantinov *et al.* (2004) found *Ruminococcus*-like species in the feces of pigs fed diets containing fibers (SBP and FOS), but not in pigs fed a control diet, suggesting that these bacteria may play a role in the utilization of DF. Moreover, there was a specific response of a novel and abundant *Lactobacillus amylovorus*-like phylotype to dietary oligosaccharides in the gut of weaning pigs (Konstantinov *et al.*, 2004). Similarly, Owusu-Asiedu *et al.* (2006) reported increased *Bifidobacteria* spp. and *Enterobacteria* spp. populations in the ileal digesta of growing pigs fed diets supplemented with guar gum or cellulose to a standard diet.

The effects of NSP compounds such as *β*G and AX have mainly been studied in isolated form, whereas in swine diets, these compounds are present as part of the grain matrix. The fermentation rate of these CHO in the intestinal tract will thus depend on their composition, form and physical properties (Le Goff *et al.*, 2003). As a consequence, cereal NSP in isolated form or within a matrix may act differently in the GIT. In this respect, Pieper *et al.* (2008) conducted an experiment with weaned pigs and found that hulless barley varieties with high soluble NSP content favored xylan- and *β*G-degrading bacteria, whereas *β*G-supplemented hulled barleys favored *Lactobacilli* spp. Moreover, there was a decrease in the number of *Lactobacilli* spp. in the ileum of pigs fed hulless/high *β*G barley-based diets. This suggests that both type and form of *β*G affect the bacterial population in the pig intestines. Processed fibers are also found to exert a positive response on health-promoting characteristics. As an example, oat fiber and *β*G isolates, fermented ropy oat-based products containing both native and microbial *β*G, stimulated *Bifidobacteria* spp. in the GIT apart from other health benefits like reduced blood cholesterol level to host (Martensson *et al.*, 2005). Like the NSP, RS is also found to affect the bacterial population in the pig intestines. Brown *et al.* (1997) found higher *Bifidobacteria* spp. counts in feces of pigs fed with a high amylose cornstarch diet, than in feces of pigs fed with a low amylose cornstarch. Part of the high amylose corn-starch becomes RS, which is fermented in the large intestine and exerts prebiotic effects.

#### Effect on gut health

The maintenance of gut health is complex phenomenon and relies on a delicate balance between the diet, the commensal microflora and the mucosa, including the digestive epithelium and the mucus overlying the epithelium (Montagne *et al.*, 2003). DF plays an important role in the function of the pig GIT. It is evidenced by several studies reporting the positive role of DF in controlling bacterial infections, particularly reducing post-weaning diarrhea (Williams *et al.*, 2001; Mateos *et al.*, 2006; Molist *et al.*, 2014), which is a major problem for the pig industry in many parts of the world. In this respect, Lizardo *et al.* (1997) reported improved digestive function in 39-day-old pigs when 12.0% SBP was included in the diet. Probably, soluble fiber sources such as SBP, are easily fermentable by the microflora in the large intestine, which could help to create a stable environment within the GIT reducing the incidence of post-weaning diarrhea. Thomsen *et al.* (2007) observed that the inclusion of DF, like fructan-rich chicory roots and sweet lupins completely protected against the development of swine dysentery. Similarly, the inclusion of DF in piglet diets enhanced intestinal populations of *Lactobacilli* spp. and reduced the incidence and severity of diarrhea (Edwards, 1996). These studies support the view that diets supplemented with fiber can protect pigs against swine dysentery. However, there is continuous debate on whether fiber exerts beneficial or detrimental effects on the development of post-weaning enteric dysentery. Pluske *et al.* (2003) noted increased incidence of clinical swine dysentery in growing pigs and diarrhea in weanling pigs fed with diets high in fermentable NSP and RS. Similar negative effect on gut health was also noticed with supplementation of isolated soluble fiber. Nursery pig diets supplemented with 0.025% *β*G increased growth performance but also increased the susceptibility to *Streptococcus suis* infection (Dritz *et al.*, 1995). These authors suggest that a complex interaction exists between growth performance and disease susceptibility in pigs fed *β*G.

There is some information available on fractions of DF of other grains like peas, chickpeas, faba beans and lupins and their effect on microbial population and gut health. Queiroz-Monici *et al.* (2005), while working with rats, found that peas and chickpeas have a bifidogenic effect due to the DF and RS present in these legumes. RS is considered a good source of butyrate production, which is utilized by the colonic cells as fuel, thus strengthening the first line of defense in the gut (Topping and Clifton, 2001). Thus, RS sources with the potential to stimulate butyrate production have a potential to improve gut health.

It can be summarized that the presence of fiber in the gut significantly affects the gut microbial environment, creates more favorable lumen conditions for gut health by stimulating the growth of ‘beneficial bacteria’ at the cost of ‘harmful bacteria’, with the possibility of some negative impact on gut health, which depends on the type of fiber substrate available for fermentation. However, there is no straightforward answer of the benefits of DF on gut health and direct evidence for enhanced resistance to unfavorable conditions is still lacking.

## Conclusion

There is wide variation found in the composition of DF in different feeds and feed ingredients, which has a major impact on their physico-chemical properties and digestion and fermentation characteristics in the GIT. In general, DF affects nutrients and energy digestibility in pigs negatively but with differences between the different types: (1) soluble DF fractions are fermented faster than insoluble fractions, produce higher amounts of VFAs and lower ammonia concentrations; (2) some DF constituents, for example RS, may stimulate butyrate production to a higher degree than others thereby contributing to an improved gut health; and (3) some DF constituents may exert ‘prebiotic effects’, enhancing ‘beneficial bacteria’ at the cost of ‘harmful bacteria’ in the pig gut. Therefore, strategic selection of DF in diets can be used as a nutritional strategy to modulate the intestinal health of swine.
